# A comparison of respiratory function in pigs anaesthetised by propofol or alfaxalone in combination with dexmedetomidine and ketamine

**DOI:** 10.1186/s13028-020-0512-y

**Published:** 2020-03-12

**Authors:** Andreas Lervik, Simen Forr Toverud, Randi Krontveit, Henning Andreas Haga

**Affiliations:** 1 Department of Companion Animal Clinical Sciences, Faculty of Veterinary Medicine, Norwegian University of Life Sciences, Ullevålsveien 72, 0454 Oslo, Norway; 2 Animal Health and Welfare Branch, Veterinary Inspectorate, Norwegian Armed Forces Joint Medical Services, Forsvarsvegen 75, 2058 Sessvollmoen, Norway; 3 HTA and Reimbursement, Norwegian Medicines Agency, Grensesvingen 26, 0663 Oslo, Norway

**Keywords:** Alfaxalone, Dexmedetomidine, Oxygenation, Pigs, Propofol, Respiratory function, TIVA, Ventilation

## Abstract

**Background:**

General anaesthesia in pigs maintained with intravenous drugs such as propofol may cause respiratory depression. Alfaxalone gives less respiratory depression than propofol in some species. The aim of the investigation was to compare respiratory effects of propofol–ketamine–dexmedetomidine and alfaxalone–ketamine–dexmedetomidine in pigs. Sixteen pigs premedicated with ketamine 15 mg/kg and midazolam 1 mg/kg intramuscularly were anaesthetised with propofol or alfaxalone to allow endotracheal intubation, followed by propofol 8 mg/kg/h or alfaxalone 5 mg/kg/h in combination with ketamine 5 mg/kg/h and dexmedetomidine 4 µg/kg/h given as a continuous infusion for 60 min. The pigs breathed spontaneously with an FIO_2_ of 0.21. Oxygen saturation (SpO_2_), end-tidal CO_2_ concentration (PE′CO_2_), respiratory rate (*f*_R_) and inspired tidal volume (V_T_) were measured, and statistically compared between treatments. If the SpO_2_ dropped below 80% or if PE′CO_2_ increased above 10.0 kPa, the pigs were recorded as failing to complete the study, and time to failure was statistically compared between treatments.

**Results:**

Alfaxalone treated pigs had significantly higher respiratory rates and lower PE′CO_2_ than propofol treated pigs, with a *f*_R_ being 7.3 /min higher (P = 0.01) and PE′CO_2_ 0.8 kPa lower (P = 0.05). SpO_2_ decreased by 0.6% and *f*_R_ by 1.0 /min per kg increase in body weight in both treatment groups. Three of eight propofol treated and two of eight alfaxalone treated pigs failed to complete the study, and times to failure were not significantly different between treatments (P = 0.75).

**Conclusions:**

No major differences in respiratory variables were found when comparing treatments. Respiratory supportive measures must be available when using both protocols.

## Background

Anaesthetised pigs are used as live tissue models in several countries for the training of prehospital trauma care providers [1]. Under these circumstances maintenance of anaesthesia with injectable anaesthetics might be necessary, as the provision of inhalational anaesthetic drugs is not possible, or even unwanted due to their effects on cardiovascular function [2]. This might also be the case for pigs used in laboratory investigations. Some of the commonly used intravenous anaesthetic drugs do however influence respiratory function when used for induction and maintenance of general anaesthesia [3], causing hypoventilation, respiratory acidosis and potentially hypoxemia.

In a previous publication, a total intravenous anaesthetic (TIVA) protocol containing propofol, ketamine and dexmedetomidine was found to provide stable cardiovascular conditions and excellent antinociception in healthy pigs [4]; features that are important when delivering anaesthesia during invasive surgical procedures in experimental animals. The effect on spontaneous ventilation was not examined in that study, but propofol has long been known to cause hypoventilation or even apnoea in several animal species including dogs and sheep when given intravenously [5, 6]. The effects of propofol on ventilation and oxygenation in spontaneously breathing pigs are only sparsely described in the literature, with apnoea and respiratory depression reported in some animals [7–9].

Alfaxalone, a steroid anaesthetic, has been used for induction and maintenance of anaesthesia in pigs without causing apnoea after induction, but hypoventilation can result when it is used for maintenance [10, 11]. At lower doses when alfaxalone was combined with isoflurane and dexmedetomidine little effect on respiration was found in spontaneously breathing pigs, with mild hypercapnia as the major finding [12]. When used to maintain anaesthesia in cats during ovariohysterectomy, alfaxalone was shown to have less effect on respiration when compared to propofol [13], but when a similar comparison was made in canine clinical patients hypoventilation occurred with both drugs [14]. To our knowledge, no comparison has been made between propofol and alfaxalone regarding their effects on ventilation and oxygenation in pigs.

The primary aim of the current study was to determine if pigs anaesthetised with either propofol–ketamine–dexmedetomidine or alfaxalone–ketamine–dexmedetomidine could maintain adequate respiratory function when spontaneously breathing atmospheric air.

## Methods

### Animals

Sixteen mixed breed (Norwegian land race 50% and Duroc 50%) pigs, 10 castrated males and 6 females, with a median (range) age of 66 (52–73) days and a mean (SD) body weight of 24.9 (4.2) kg in the propofol group and 25.9 (3.7) kg in the alfaxalone group were included. The pigs were acquired from the same breeder and identified using their existing ear tag numbers. They were transported to the research animal facility of the Norwegian University of Life Sciences where they were housed for approximately 14 days prior to the experiment. All pigs were allowed to follow normal light–dark cycles in a room with natural day light and a room temperature kept between 15 and 20 ℃. They were fed a commercial pig diet in combination with free access to hay. Approximately 1 week prior to this experiment the pigs were used in another anaesthesia study and a minimum wash out period of 7 days was allowed between experiments. Their health status was monitored minimanual breaths in the post induction period duemum once daily during the entire period. The study was approved by the Norwegian National Animal Research Authority (FOTS 14,277).

### Study design

A balanced, randomized study design was used. The 16 pigs were randomized in blocks of four to receive either propofol–ketamine–dexmedetomidine or alfaxalone–ketamine–dexmedetomidine by drawing paper notes. None of the investigators were blinded to the given treatment.

### Anaesthesia, monitoring and data collection

Food, but not straw and water, was withheld for approximately 12 h before premedication, and all pigs were found healthy based on a clinical examination before each experimental session. Their body weight was measured on the day of the experiment.

Premedication was given using ketamine 15 mg/kg (Ketamine Le Vet 100 mg/mL; Le Vet Beheer B.V., TV Oudewater, Holland) in combination with midazolam 1 mg/kg (Midazolam 5 mg/mL; B. Braun, Melsungen, Germany) administered intramuscularly (IM) in the cervical muscles. After sedation an intravenous catheter (Venflon Pro; Becton Dickinson Infusion Therapy, Franklin Lakes, NJ, USA) was placed in an auricular vein. The pigs were preoxygenated for 4–5 min with 100% O_2_ delivered by a face mask, and anaesthesia was thereafter induced by slow intravenous titration of propofol (Propofol–Lipuro 20 mg/mL; B. Braun) or alfaxalone (Alfaxan 10 mg/mL; Jurox, Rutherford, Australia) to allow endotracheal intubation after application of topical lidocaine (Xylocain 100 mg/mL spray; Aspen, Dublin, Ireland). The induction of anaesthesia was always performed by the same investigator (AL), and the total dose needed to allow endotracheal intubation and the internal diameter of the endotracheal tube (Rüschelit super safety clear, Teleflex Medical, Westmeath, Ireland) was noted. The cuff was inflated to a pressure of 40–45 cm H_2_O using a manometer syringe (AG Cuffill, Hospitech Respiration, Kfar Sabam, Israel).

After intubation, the pigs were placed in left lateral recumbency, and allowed to breathe atmospheric air spontaneously. Upper airway suction through the endotracheal tube was allowed if excessive airway secretion was detected by auscultation. In addition, single manual breaths with a FIO_2_ of 0.21 were allowed to be administered with a manual resuscitation bag (Laerdal Silicone Resuscitator, Laerdal Medical, Stavanger, Norway) if the SpO_2_ dropped below 80% during the first 5 min after intubation. Anaesthesia was maintained with undiluted propofol 8 mg/kg/h or undiluted alfaxalone 5 mg/kg/h in combination with ketamine diluted to 50 mg/mL at 5 mg/kg/h and dexmedetomidine (Dexdomitor 0.5 mg/mL, Orion Corporation, Esbo, Finland) diluted to 50 µg/mL at 4 µg/kg/h intravenously (IV). All drugs were diluted using 0.9% NaCl (Natriumklorid Fresenius Kabi, Fresenius Kabi, Halden, Norway) and were given as a continuous infusion delivered by syringe drivers (Alaris GH Plus, BD Medical, Franklin Lakes, NJ, USA). The doses of propofol, ketamine and dexmedetomidine were chosen based on the results from a previous study [4]. The alfaxalone dose used was based on a dose titration pilot study in four pigs, where the lowest infusion rate abolishing the motor response to the same standardised electrical nociceptive stimulation used in the previous study [4] was determined.

The pigs were monitored using a multiparameter anaesthetic monitor (GE Carescape Monitor B650; GE Healthcare, Helsinki, Finland). Heart rate (*f*_H_), respiratory rate (*f*_R_) based on the capnography trace, 3-lead electrocardiogram, oxygen saturation (SpO_2_), end tidal CO_2_ (PE′CO_2_), fractioned inspired oxygen concentration (FIO_2_), and oesophageal temperature were recorded and automatically downloaded every 5 s for 60 min using data collection software (iCollect Version 5.0, GE Healthcare). SpO_2_ was measured using a pulse oximetry finger sensor (TruSignal finger sensor, GE Healthcare) placed on the lateral digit of right hind- or front limb. The plethysmography trace was continuously inspected by one of the examiners to ensure proper signal quality. If a flattening of trace or a sudden drop in SpO_2_ was observed the probe was repositioned, and the trace was assessed again. In addition, a pitot tube with a sample port for gas monitoring (Pedi Lite + Flow Sensor, GE Healthcare) was fitted to the end of the endotracheal tube and connected to the monitor using the manufacturers tubing (Spirometry tube, disposable, yellow, GE Healthcare). The capnography trace was continuously inspected by one of the examiners. According to the manufacturer, the pitot tube allows measurements of tidal volumes from 5 to 300 mL. Inspired tidal volumes (V_T_) were recorded every 5 s for 60 min.

If the SpO_2_ dropped below 80% for more than 30 s, FIO_2_ was increased to 0.5. Similarly, if PE′CO_2_ increased above 10.0 kPa for more than 30 s, intermittent positive pressure ventilation (IPPV) was instituted for the remaining study period. In either case, the pigs were then recorded as failing to complete the study period, and time to failure was noted. An SpO_2_ of 80% represents severe hypoxemia and was chosen as our cut off value for intervention. Using the alveolar oxygen equation and the oxyhaemoglobin dissociation curve for adult miniature pigs with a P50 of 4.30 kPa [15] this SpO_2_ would result from hypoventilation leading to a PaCO_2_ of approximately 10.5 kPa in a lung with normal gas exchange.

All pigs received a balanced electrolyte solution (Ringers acetate; Fresenius Kabi) intravenously at a rate of 0.33 mL/kg/h delivered by a volumetric infusion pump (Volumat Agilia; Fresenius Kabi). In addition, the total volume of drugs infused was 1.08 mL/kg/h, with a total infusion rate throughout the study of 1.41 mL/kg/h. All fluid and drug infusions were administered through the same intravenous catheter placed in the auricular vein. In addition, the pigs were covered with bubble wrap to avoid heat loss and external heat was provided with a forced air patient warming device (Bair Hugger, 3 M, St. Paul, MN, USA) as long as the body temperature was < 39.5 ℃. After completing a second study performed under the same anaesthetic the pigs where euthanised with pentobarbital (Euthasol vet 400 mg/mL, Le Vet Beheer) given intravenously.

### Evaluation of anaesthetic depth and electroencephalography

Sixty-five minutes after intubation a standardised evaluation of anaesthetic depth was performed. Clinical signs of anaesthetic depth were scored by the same investigator as follows: First Eye position (ventral = 0, central = 1), nystagmus (present = 0, absent = 1), palpebral reflex (present = 0, absent = 1), and corneal reflex (present = 0, absent = 1) were assessed. Thereafter mechanical nociceptive stimulation was applied to the lateral dewclaw of the right front limb by a latex-coated forceps with a clamping area 1 × 1 cm. The applied clamping pressure was monitored with a spring balance attached to one forceps arm at a point an equal distance from, and on the opposite side of the articulation as the clamping jaws. The force applied at clamping was 100 N and maximum clamping time for each stimulus was 59 s. Stimulation was stopped at withdrawal of the limb or if vigorous movement in other limbs or whole-body movement was observed. The withdrawal was scored as present (= 0) or absent (= 1). A summarised score ranging from 0 to 5 for eye reflexes, eye position and response to clamping was noted.

In seven pigs, electroencephalography (EEG) was recorded. For recording of a two-channel referential EEG needle electrodes (Aiglette, Technomed Europe, Maastricht, Netherlands) were used. Electrodes were placed 1 cm caudal to the lateral angle of the eye and 1 cm medial to the temporal line bilaterally, and these electrodes were referred to an electrode placed in the median plane 2 cm caudal to the recording electrodes. A ground electrode was placed caudal to the atlas wing. The resistance of each electrode pair was kept below 3 kΩ. The electrodes were connected to an EEG monitor (A-1000™, Aspect Medical Systems, Newton, MA, USA). The monitor filters were set as follows—high frequency filter: 50 Hz, 50/60 Hz filter: 50 Hz and low frequency filter: 2.0 Hz. The monitor automatically detected burst suppression and calculated the percentage of epochs in the previous 63 s where the EEG signal was considered suppressed. This percentage is called burst suppression ratio (BSR) and was updated every 5 s.

### Statistical analysis

Respiratory data collected from 5 min (time point 0) to 65 min (time point 60) after intubation were used for statistical analysis, with a total observation period of 60 min. V_T_ was indexed to body weight for statistical analysis (V_T_/kg).

For graphical evaluation and a Kaplan–Meier analysis, a database was created in JMP 14.1.0 (SAS Institute Inc., Cary, NC, USA). The mean SpO_2_, PE′CO_2_, *f*_R_ and V_T_/kg for each minute was calculated and used for graphical evaluation of respiratory parameters. In addition, time to failure was compared between treatments using a Kaplan–Meier analysis including a log-rank test.

Further statistical comparison of respiratory parameters was performed using Stata SE 15 (Stata Corp LLC, Lakeway Drive College Station TX., USA). The data collection time points were reduced to recordings every 10 min, where the mean for 1 min at each evaluation time point was calculated. Seven means per subject (at 0, 10, 20, 30, 40, 50 and 60 min from baseline) were included in the analysis. Distribution of the outcome variables SpO_2_, PE′CO_2_, *f*_R_ and V_T_/kg were assessed using histograms. Data sampled after a pig failed the study were handled using “last observation carried forward” (LOCF). At time points where FIO_2_ was increased to 0.5 due to low SpO_2_, a SpO_2_ of 79% was used when analysing the data. Similarly, at time points where IPPV was used, PE′CO_2_ was set to 10.1 kPa; in addition, the last *f*_R_ and V_T_/kg recorded before instituting IPPV were also used. Missing data were otherwise not imputed. Linear mixed models with individual pig as random effect and a compound symmetry (exchangeable) correlation structure were fitted for all outcome variables to assess the impact of treatment on the outcome in question. Other correlation structures were considered, but not deemed feasible. In the present data with the low sample size, a better fit of the unstructured covariance structure came at a cost of using many degrees of freedom. In addition, the other common matrices would result in overfitting due to the fact that the random effect was at the same level as the repeated measures (pig). The correlation and covariance structure observed in the data supported the compound symmetry. Sex and body weight were included as fixed effects in all models.

Intraclass correlation coefficients (ICC) were calculated based on the variance estimates from the models to give an estimate of the level of clustering in the data.

$${\text{ICC}}\; = \;\sigma ^{{\text{2}}} _{{{\text{pig}}}} \;/\;(\sigma ^{{\text{2}}} \, + \,\sigma ^{{\text{2}}} _{{{\text{pig}}}} ).$$

Models with and without pig random effect were compared with likelihood ratio tests (LRT). Assumptions for linear mixed models were evaluated as described in the literature. For all statistical tests, an alpha of 5% was used.

### Results

The mean (SD) dose needed to allow sufficient anaesthetic depth for intubation was 2.54 (0.80) and 1.10 (0.23) mg/kg in the propofol and alfaxalone group respectively. Single manual breaths in the post induction period due to low SpO_2_ suggesting possible hypoxemia were necessary in two of eight pigs in each group. None of the pigs experienced periods of apnoea in this period.

**Fig. 1:**
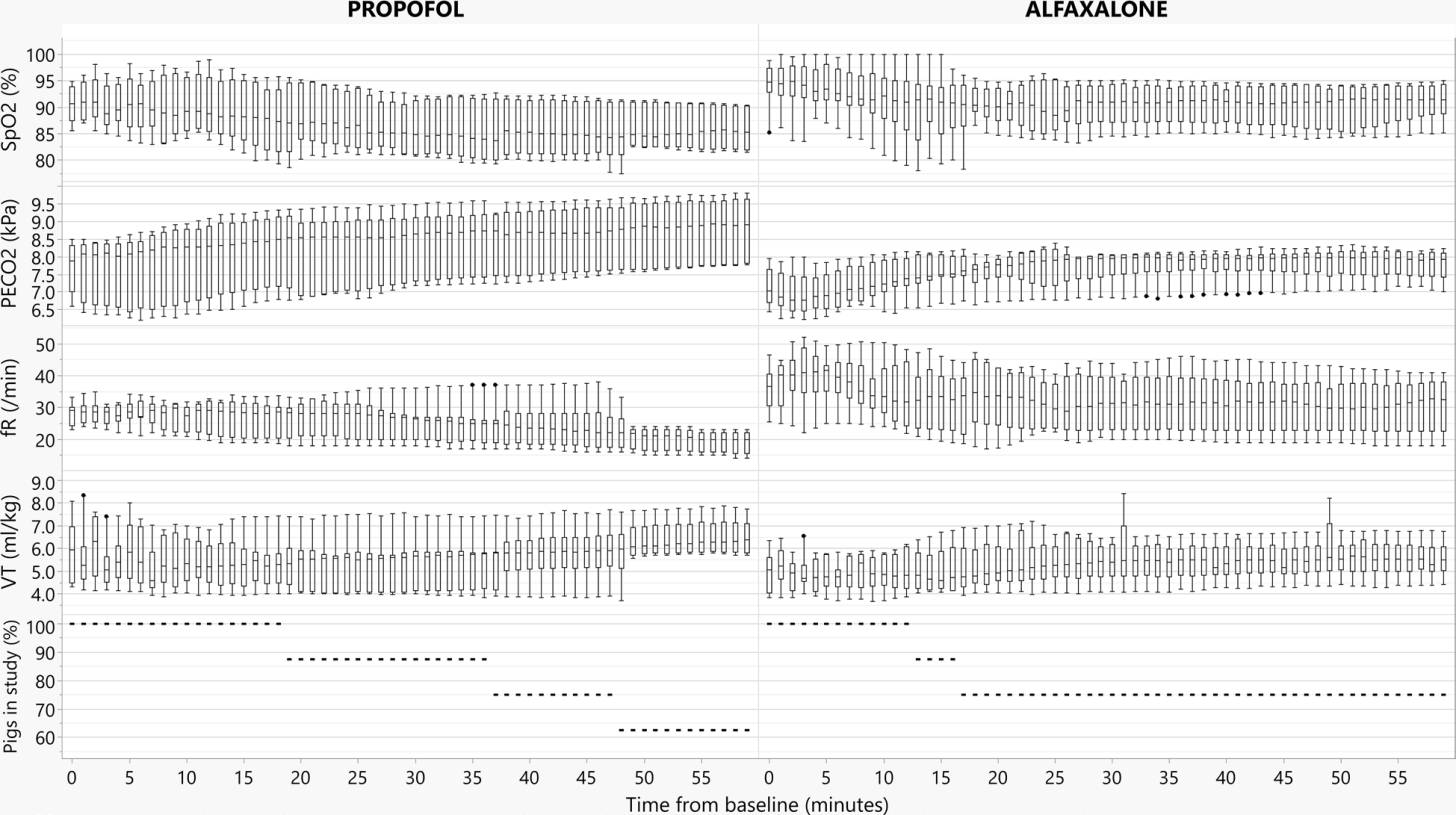
Box and whisker plot showing the median, interquartile range and range for SpO_2_, PE′CO_2_, *f*_R_, V_T_/kg calculated as the mean for each minute plotted against time in anaesthesia for pigs given either propofol 8 mg/kg/h or alfaxalone 5 mg/kg/h in combination with ketamine 5 mg/kg/h and dexmedetomidine 4 µg/kg/h. In addition is the percentage of pigs remaining in the study of the initial eight included in each group displayed (dotted line)

Measured respiratory parameters for the entire study period and time to failure are summarised in Fig. 1. Three of eight propofol treated and two of eight alfaxalone treated pigs failed to complete the study. All failures where due to low SpO_2_ and respiratory parameters at the time of failure are summarised in Table 1. In two propofol treated and one alfaxalone treated pigs IPPV was also necessary due to PE′CO_2_ > 10 kPa after increasing FIO_2_ to 0.5. Results of the Kaplan Meier analysis did not reveal any significant difference in time to failure between treatments (P = 0.750).

**Table 1:** Respiratory parameters at time point of study failure for pigs failing to complete the study are shown. The pigs are anaesthetised with either propofol 8 mg/kg/h or alfaxalone 5 mg/kg/h in combination with ketamine 5 mg/kg/h and dexmedetomidine 4 µg/kg/h

Treatment	Pig no	Time to failure (min)	SpO_2_ (%)	PE′CO_2_ (kPa)	V_T_/kg (mL/kg)	*f*_R_ (/min)
Propofol	1	48	77	8.6	3.7	34
	4	37	79	8.9	3.8	25
	7	19	79	9.3	6.4	20
Alfaxalone	3	13	78	8.2	6.7	23
	6	17	79	8.2	5.2	22

Results from the mixed models are shown in Table 2. The results from the mixed models were consistent with the descriptive statistics. Pigs treated with alfaxalone had a higher respiratory rate (P = 0.01) and lower PE′CO_2_ (P = 0.05) than propofol treated pigs, with a *f*_R_ that was 7.3 /min higher and a 0.8 kPa lower PE′CO_2_ for the study period. Body weight had statistically significant impact on SpO_2_ and *f*_R_ in both treatment groups, with SpO_2_ decreasing 0.6% and *f*_R_ decreasing 1.0 /min per kg increase in body weight.

**Table 2:** Results from the linear mixed model analysis on effect of treatment on SpO_2_, PE′CO_2_, *f*_R_ and V_T_ in pigs anaesthetised with either propofol 8 mg/kg/h or alfaxalone 5 mg/kg/h in combination with ketamine 5 mg/kg/h and dexmedetomidine 4 µg/kg/h (n = 16)

Variable and level	Estimate (SE)	P-value	95% Confidence interval
SpO_2_			
Treatment			
Propofol	Baseline	–	–
Alfaxalone	3.6 (2.3)	0.12	− 0.87, 8.04
Sex			
Male	Baseline	–	–
Female	3.4 (2.3)	0.14	− 1.13, 8.02
Body weight	− 0.6 (0.3)	0.04	− 1.20, − 0.05
PE′CO_2_			
Treatment			
Propofol	Baseline	–	–
Alfaxalone	− 0.8 (0.4)	0.05	− 1.62, 0.01
Sex			
Male	Baseline	–	–
Female	− 0.6 (0.4)	0.17	− 1.43, 0.25
Body weight	0.1 (0.05)	0.14	− 0.03, 0.19
*f*_R_			
Treatment			
Propofol	Baseline	–	–
Alfaxalone	7.5 (3.1)	0.01	1.51, 13.51
Sex			
Male	Baseline	–	–
Female	2.3 (3.1)	0.47	− 3.91, 8.41
Body weight	− 1.0 (0.4)	0.02	− 1.74, − 0.18
V_T_/kg			
Treatment			
Propofol	Baseline	–	–
Alfaxalone	0.0 (0.5)	0.94	− 1.0, 1.0
Sex			
Male	Baseline	–	–
Female	0.1 (0.5)	0.80	− 0.9, 1.2
Body weight	− 0.1 (0.1)	0.235	− 0.2, 0.1

From the linear mixed models calculated ICCs were very high; 53.8% for SpO_2_, 57.4% for PE′CO_2_, 59.7% for *f*_R_ and 76.8% for V_T_/kg. Results from the LRT gave P-values < 0.0001 for all four models.

The median (range) summarised score for anaesthetic depth after 60 min was 4 (3–4) and 4 (2–5) in the propofol and alfaxalone group respectively. One of eight and three of eight pigs had a positive withdrawal reflex in response to clamping of the dewclaw in the propofol and alfaxalone group respectively. EEG was recorded in four pigs in the propofol and three pigs in the alfaxalone group. BSR was 0, 0, 2 and 9% and 0, 3 and 33% in the respective group.

## Discussion

Pigs anaesthetised with propofol–ketamine–dexmedetomidine or alfaxalone–ketamine–dexmedetomidine both displayed compromised respiratory function when breathing atmospheric air, with 37.5% and 25% respectively needing intervention based on our predetermined cut-off levels. The cut off levels chosen in this study were on purpose set rather liberally to avoid exclusion of too many subjects during the study period. The time point of failure was evenly distributed over time in both treatment groups. When using these anaesthetic regimes in pigs, supplemental oxygen must be given, as moderate to severe hypoxemia as detected by pulse oximetry was the main cause of failure to complete the study. Even if none of the pigs experienced apnoea, moderate to severe hypoventilation was observed, and the ability to provide intermittent positive pressure ventilation should be available if using these anaesthetic regimes.

It is likely that the main mechanism of hypoxemia in our study is anaesthesia-induced hypoventilation. Our study was not designed to elucidate the pathophysiological mechanisms of hypoxemia that we observed, but rather to characterise the respiratory function using a particular anaesthetic regime in healthy pigs. After performing a theoretical calculation of the alveolar partial pressure of oxygen when breathing atmospheric air in the anaesthetised pigs, and taking the published P50 for adult miniature pigs [15] and a right shift of the oxyhaemoglobin dissociation curve due to hypercapnia into account, it seems that the drop in SpO_2_ is clearly associated with the observed increase in PE′CO_2_. This is also supported by the physiological parameters at the time of failure, where moderate to severe hypercapnia is observed in combination with hypoxemia in all pigs. The registered tidal volume was also very low in two of the pigs at this time point, with the likelihood of a high Pa-E′CO_2_-difference, supporting that the PaCO_2_ is even higher than the observed PE′CO_2_. In addition to this, a study in healthy pigs showed that the degree of venous admixture is low during spontaneous breathing, making this a less likely cause, but a direct comparison to our animals is difficult as these animals where anaesthetised with ketamine, placed in dorsal recumbency and received 100% oxygen [16].

In the current study, a statistically significant difference between treatments was found for respiratory rate and PE′CO_2_. This stands in contrast to a clinical study in dogs, where no significant differences were found in respiratory rate, tidal volume or PE′CO_2_ when comparing TIVA with propofol and alfaxalone [14]. A relatively high respiration rate was also found in previous studies in pigs when anaesthesia was induced or maintained with alfaxalone [10, 17]. In a systematic review comparing respiratory rate in dogs and cats after induction with either alfaxalone or propofol no evidence of difference could be found between the two agents [18]. The PE′CO_2_ in our study was lower in the alfaxalone treated than in propofol treated pigs. The 95% confidence interval for PE′CO_2_ and the descriptive data also supports that a population of pigs anaesthetised with alfaxalone–ketamine–dexmedetomidine likely will maintain a better alveolar minute ventilation than pigs receiving propofol–ketamine–dexmedetomidine, but the magnitude of this difference might not be of clinical importance. LOCF was used in the statistical comparison in this study, and this approach can be debated. It is traditionally viewed upon as a conservative approach, but this will of course depend on the true missing values [19]. It is plausible that the imputed data in our study represent a truly conservative picture of the reality, and that the difference in PE′CO_2_ could have been larger without our intervention at a PE′CO_2_ of 10 kPa.

Arterial blood gas analysis can be considered a gold standard when investigating the effectiveness of alveolar ventilation and gas exchange in the clinical setting [20]. Arterial catheterisation after induction was not performed, as arterial catheterisation would have delayed the documentation of the respiratory effects by several minutes. In addition, the access to patent arteries were important for the second study performed in the same pigs. As such, a major weakness in the current investigation was the use of pulse oximetry, capnography and spirometry as the sole methods for the assessment of oxygenation and alveolar ventilation. These methods have their limitations when it comes to both accuracy and precision [21, 22]. Several studies in human patients compare the performance of pulse oximetry to arterial oxygen saturation (SaO_2_) showing a varying accuracy, but also that the accuracy is higher in the range from 80–100% [23, 24]. The precision and accuracy of pulse oximetry in newborn piglets has been examined, using an ear clip sensor placed on the thigh. The investigators found a deviation from measured SaO_2_, in particular with values below 60% and during periods where hypoperfusion was suspected [25]. The saturations measured in the current study were all in the range from 80–100%. In addition, none of the pigs experienced hypoperfusion or hypothermia that could have influenced pulse oximeter performance. Pulse oximetry plethysmography curves and capnography waveforms were continuously assessed during data sampling in this study to reduce the risk of inaccurate measurements.

A tidal volume of 9 mL/kg has been reported in awake, young tracheotomised pigs [26]. In our study, observed tidal volumes were similar between groups, but lower than reported in awake pigs. Anatomical dead space is a constant factor, and a relatively larger proportion of each breath would be dead space if the tidal volume decreases. This could easily lead to variations in the measured PE′CO_2_ that does not correspond to the true PaCO_2_, and thus not reflect the true alveolar ventilation. In addition, one could argue that the higher *f*_R_ in the alfaxalone treated animals could influence the measured PE′CO_2_, introducing a bias towards lower values [27]. The V_T_/kg is however very similar in the two groups, thus supporting that alveolar ventilation was higher in the alfaxalone group. Single manually performed positive pressure ventilations could have been performed to reduce the influence of dead space ventilation on the PE′CO_2_. In the authors experience this would however influence the respiratory pattern of the pigs, and potentially the study result. Despite limitations in the methodology, we are confident that the comparison between treatments and the main conclusion on the suitability of the TIVA protocols in spontaneously breathing pigs is valid. In addition, pulse oximetry and capnography are the commonly used monitoring modalities for clinical decision-making during anaesthesia for live tissue training in pigs. Interpretation of absolute values should on the other hand be made with caution.

Body weight had significant impact on SpO_2_ and *f*_R_ in our study. One possible explanation for the drop in SpO_2_ could be that heavier pigs reached a deeper plane of anaesthesia during the study period, as the body weights in the study population varied from 20 to 31 kg. Increasing body weight could result in higher drug plasma concentrations, and thereby negative impact on respiratory function. An allometric dose effect of medetomidine in pigs has previously been observed by the authors, with heavier animals becoming more sedated than pigs with lower body weight when receiving 80 µg/kg intramuscularly [28], however an effect of age cannot be excluded. In humans given an infusion of 5 mg/kg/h of propofol plasma concentrations increase with bodyweight, also when corrected to lean body weight [29]. Repeated measurements of anaesthetic depth could have elucidated the temporal change in anaesthetic depth during the examination period in this study. Nociceptive stimulation could however potentially influence the pattern of spontaneous ventilation, and the authors decided to delay the assessment of anaesthetic depth to the end of the study period. All pigs were induced to a sufficient depth to allow endotracheal intubation at the start of the study, and the median score for anaesthetic depth was the same in both groups at the end of the study period. We therefore believe that the anaesthetic depth was similar in both groups in the study period.

When comparing the effect of anaesthetic drugs on physiological variables, similar anaesthetic depth should be presumed. However, establishing equipotent doses of anaesthetic drug combinations can be very challenging. At the same time, determining anaesthetic depth is difficult, with physical signs including ocular reflexes, cardiovascular and respiratory response and response to nociceptive stimulation being commonly utilized in clinical veterinary anaesthesia. The median score for anaesthetic depth in the current study was the same in the two groups, with a somewhat larger range in both directions in the alfaxalone group. The score was based on what is typically used during clinical anaesthesia in pigs, with the exception of arterial blood pressure response to nociceptive stimulation that was not evaluated. Arterial cannulation was not performed for reasons mentioned above. Several of the pigs in both treatment groups displayed burst suppression at the evaluation time point after 60 min, leading us to conclude that a relatively profound level of cerebrocortical depression was present [30]. Interestingly, a previous study in isoflurane anaesthetised pigs also shows that burst suppression can be present in the EEG of pigs responding with movement to nociceptive stimulation [31]. In the current study, pigs in both groups moved in response to mechanical nociceptive stimulation. Based on the burst suppression seen in some pigs one could argue that this is probably not a conscious response, but rather spinally mediated. Muscle relaxation and immobility are however important characteristics of general anaesthesia, and it is possible that a dose adjustment must be made when using these anaesthetic protocols during surgery.

The number of animals included was not based on a previous sample size calculation for this study, rather than on the number of animals needed for another experiment performed under the same anaesthesia. This is not according to the ARRIVE guidelines for animal experimentation [32]. This study may be underpowered, and other statistically significant differences in the examined respiratory parameters might have been found if more animals had been included. The authors still find it highly unlikely that the overall conclusion on the effect of the anaesthetics regimes on respiration would have been different.

## Conclusions

The current study shows that pigs anaesthetized with propofol–ketamine–dexmedetomidine or alfaxalone–ketamine–dexmedetomidine can experience hypoxemia and hypercapnia, and that proper supportive measures must be available when using these anaesthetic regimes. Possible allometric dose effects of the drugs used should be considered and warrants further investigation.

## Data Availability

The datasets generated during and/or analysed during the current study are available from the corresponding author on reasonable request.
